# The treatment zone decentration and corneal refractive profile changes in children undergoing orthokeratology treatment

**DOI:** 10.1186/s12886-022-02396-w

**Published:** 2022-04-18

**Authors:** Weiping Lin, Tianpu Gu, Hua Bi, Bei Du, Bin Zhang, Ruihua Wei

**Affiliations:** 1 Tianjin Key Laboratory of Retinal Functions and Diseases, Tianjin Branch of National Clinical Research Center for Ocular Disease, Eye Institute and School of Optometry, Tianjin Medical University Eye Hospital, Tianjin, 300384 People’s Republic of China; 2 College of Optometry, Nova Southeastern University, Davie, FL 33314 USA

**Keywords:** Orthokeratology, Treatment zone decentration, Corneal refractive power, Myopia control

## Abstract

**Background:**

To confirm the association between treatment-zone (TZ) decentration and axial length growth (ALG) in children who underwent orthokeratology; and to explore the association between TZ decentration and relative corneal refractive power (RCRP) profile, which was known to be significantly associated with ALG retardation.

**Methods:**

Four hundred myopic children of age 12 years participated in the study, with 200 wearing orthokeratology lenses and the other 200 wearing single-vision spectacle as the controls. Cycloplegic refraction was performed at baseline. Axial length was measured at baseline and 12 months after initial lens wear, and ALG was defined as the difference. In the ortho-k group, TZ decentration and the RCRP map were calculated from the topography map obtained at the 12-month visit. RCRP were summed within various chord radii from the cornea center, and the association to TZ decentration, spherical equivalent (SE), ALG were analyzed with linear regressions.

**Results:**

Compared to the controls, children wearing orthokeratology lenses had significantly smaller ALG over 1 year (0.1 ± 0.15 mm vs. 0.32 ± 0.17 mm, *p* < 0.001). ALG was significantly and negatively associated with summed RCRP within the central cornea of 2 mm in radius. The mean TZ decentration was 0.62 ± 0.25 mm, and the mean direction was 214.26 ± 7.39 degrees. ALG was negatively associated with the TZ decentration magnitude (*p* < 0.01), but not the direction (*p* = 0.905). TZ decentration caused an asymmetrical distribution of the RCRP with the nasal side plus power shifting towards the corneal center. For chord radius ranging 1-2 mm, the association between TZ decentration and the summed RCRP were significant, and the proportion of variance accountable increased with chord radius. For chord radius beyond 1.5 mm, the association between baseline spherical equivalent (SE) and summed RCRP was significant. The portion of variance accountable by SE increased and peaked in 2.5 mm chord radius.

**Conclusions:**

A larger TZ decentration was associated with a larger summed RCRP in the central cornea. It may be one of the possible reasons why TZ decentration is beneficial to retarding myopia progression.

## Background

An Orthokeratology lens is a rigid contact lens with a reverse geometry on its back surface [1]. Through overnight-only wear, it flattens the central portion of the cornea to correct the refractive error for a good daytime vision. It also steepens the mid-peripheral part of the cornea, which presumably induces a myopic retina defocus to retard the axial length growth (ALG). It has become one of the most effective means for myopia control [2, 3]. Compared to single-focus spectacles and contact lenses, Orthokeratology lenses reduce the ALG in adolescents by 32–55% [4, 5].

In orthokeratology lens fitting, clinicians have been long taught to have the lens perfectly centered on the corneal geometric center. However, several recent studies reported that a larger treatment zone (TZ) decentration was associated with smaller ALG [6–8]. Chen et al. [6] found that the ALG was negatively correlated with TZ decentration (*r* = − 0.147) in 101 children with orthokeratology for 24 months. Anken et al. [7] reported that the TZ decentration of orthokeratology can delay the development of myopia in a 30-children self-control study for a year. Wei et al. [8] demonstrated that a larger TZ decentration is significantly associated with smaller ALG (*r* = − 0.25) in 352 children wearing orthokeratology lenses for 12 months.

It is not clear how TZ decentration contributes to retard myopia progression. Early studies reported that a decentered TZ increases corneal asymmetry [9, 10]. Hiraoka et al. found that the amount of decentration significantly increased coma-like aberrations [11], and coma-like aberration was negatively correlated with ALG [12]. More recent studies started to quantify corneal topographic changes and explore their association with ALG in children wearing orthokeratology lenses. Yang XY et al. reported that a shorter distance between the rising edge of the steepened zone and the corneal center (X50) is significantly associated with shorter axial length growth [13]. Hu et al. reported that summed relative corneal refractive power (RCRP) shift from the baseline within the central 4-mm diameter zone is significantly correlated with axial length growth [14, 15]. Most recently, Pauné et al. reported that children treated with lenses of a smaller back optic zone diameter (BOZD) had smaller axial length growth than those treated with a lens of larger BOZD [16, 17]. Huang et al. found that a larger BOZD design(6 mm) did not show slower myopia progression than a smaller BOZD design(3 mm) in children treated with MSCL [18]. Zhang et al. found that the asphericity of the treatment zone may affect axial elongation in children undergoing ortho-k therapy [19]. These findings indicated that the size of the central area over which RCRP is summed needs to be systematically explored. Therefore, this study aimed to investigate how TZ decentration and SE were associated with summed RCRP at different chord radii.

## Methods

### Subjects

This retrospective study was conducted at the Tianjin University Eye Hospital (Tianjin, China) between August 2018 and July 2019. The study adhered to the tenets of the Declaration of Helsinki and was approved by the Institutional Ethical Committee Review Board of Tianjin Medical University Eye Hospital. Four hundred myopic children of age 12 years participated in the study, with 200 wearing orthokeratology lenses and other 200 wearing single-vision spectacle as the controls. According to the inclusion criteria below, all children were deemed suitable for this study and included for analysis. The initial inclusion criteria were: aged 12 years; SE of cycloplegic refraction from − 1.00 D to − 6.00 D; with-the-rule astigmatism less than 1.50D; best-corrected monocular visual acuity better than 20/20. Cycloplegic refraction with compound tropicamide eye drops (5 mg/mL, one drop every 5 min for four times) before fitting orthokeratology lenses or single-vision spectacles; The eye surface is healthy, and the cornea is not stained; Exclusion criteria were: strabismus or ocular surface disease, history of surgery, and contact lenses wear history.

### Orthokeratology lens fitting and follow up plan

Children were fitted with a spherical four-zone orthokeratology lens (Euclid Systems Corporation, Herndon, USA) composed of oprifocon A (Boston Equalens II) with an oxygen permeability (DK) of 127 × 10^− 11^ (cm^2^/s) (mL O_2_/mL· mmHg). Total lens diameter ranged from 10.2 to 11 mm, the back optical zone diameter was 6.2 mm, the reverse curve width was 0.5 mm, the alignment curve was between 1.0 and 1.4 mm, and the peripheral curve width was 0.5 mm. Lens fitting procedures strictly followed the guidelines provided by the lens manufacturer. Briefly, the first trial alignment curve for the lens was based on the corneal topography (Medmont, International Pty. Ltd., Victoria, Australia), flat-K, corneal eccentricity, and the horizontal iris diameter. Fitting quality was evaluated by fluorescence staining 1 h after the lens placement. A good fitting was indicated by an optical zone covering the pupil, no apparent decentration of the lens, blink lens movement less than 1 mm, and a bullseye pattern with fluorescence staining. Over-refraction was performed to determine target power plus 0.75 diopters as the final order. Children received instructions for lens-wearing and cleaning at that time. Lenses were required to be worn for more than 8 h per night. Follow-up visits were scheduled at 1 day, 1 week, 2 weeks, and 1 month after the initial lens wear and at least once per 3 months afterward. All children included were continuously worn the lenses and do a topographic map examination within 4 h after removing the lenses. All children had unaided visual acuity better than 20/25.

### Axial length measurement

Axial length was measured before ortho-k lens fitting or spectacle-wearing (baseline) and 12 months after treatment using noncontact optical biometry (Lenstar 900; Haag-Streit AG, Switzerland). Axial length growth was defined as the difference between the two measurements. The same experienced technician measured the axial length three consecutive times, and the mean value was taken for data analysis. The results met the instrument’s quality control requirements.

### Corneal topography

**Fig. 1:**
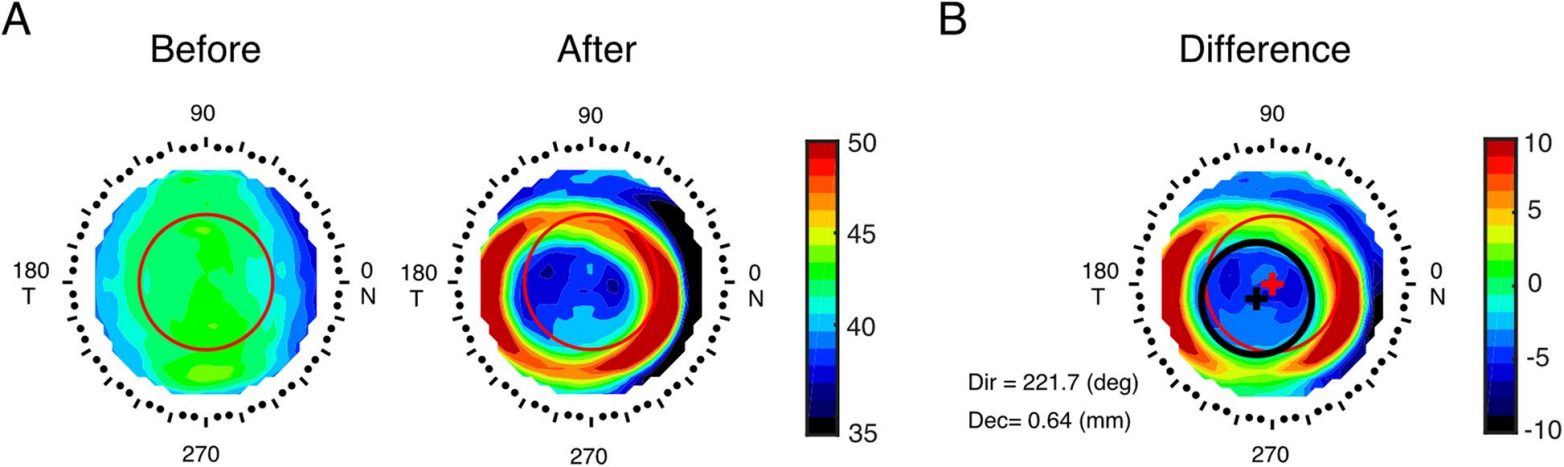
Methods to determine treatment zone decentration. **A** topographic map before and after orthokeratology treatment. **B** Method to determine TZ decentration during the 12-month follow-up visit. The red cross indicates the geometric center, the black circle represents the fitted treatment zone, and the black cross indicates the center of the treatment zone

Corneal topography was obtained with Medmont (Medmont, International Pty. Ltd., Victoria, Australia) at baseline and each follow-up visit. Three maps were acquired at each visit, and the best-focused image was used for analysis. The 12-month topographic outputs were taken as representative of the post-treatment topography in the current study. All measurements were done between 8 to 10 am to minimize the diurnal variation. For treatment zone delineation, the post-treatment tangential curvature map (Fig. 1A Right) was subtracted from the baseline map (Fig. 1A Left) to derive the difference map. The area containing locations reduced by more than 0.00 D was defined as the TZ, and its boundary was fitted to a circle using a custom MATLAB function (MathWorks, Natick, WA). (Fig. 1B) The radius between the center of the circle (black cross, Fig. 1B) and the geometric center (red cross, Fig. 1B) was defined as the TZ decentration [ 20].

**Fig. 2:**
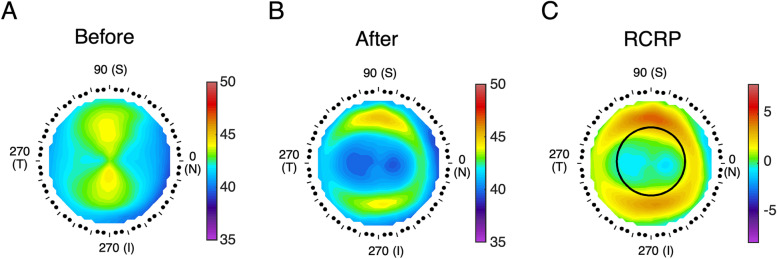
Methods to determine RCRP. **A**) Axial map at baseline, (**B**) axial map in 12 months after treatment, (**C**) The RCRP map

Axial corneal topographic maps were used to analyze the relative cornea refraction power (RCRP). The RCRP map was derived by subtracting the center value from every point on the 12-month axial map (Fig. 2). Axial maps were used to analyze the relative cornea refraction power (RCRP). The RCRP map (Fig. 2. C) was derived by subtracting the center value from every point on the 12-month axial map (Fig. 2 B). The sum was defined as the summation of the points located within a chosen chord radius.

### Statistical analysis

Only the data from the right eyes were used for statistical analysis. For descriptive purposes, the means and standard deviations were calculated for TZ decentration, spherical equivalent (SE), RCRP, and axial length growth (ALG). Simple linear regressions were used to analyze the relationships between ALG and these parameters. All statistical analyses were performed using R software (version 3.2.2 http://www.R-project.org/). A *p*-value < 0.05 value was defined as statistically significant.

## Results

There were no differences in baseline characteristics (gender ratio, axial length, and refractive error) between the ortho-k group and the spectacle group (Table 1).

**Table 1:** Baseline characteristics in two groups presented as mean ± SD

Parameters	OK group	SP group	*P* value
Gender(M/F)	113/87	104/96	0.97
Refractive error	−3.28 ± 1.54	− 3.15 ± 1.62	0.38
Axial length	25.04 ± 0.92	24.92 ± 0.88	0.16

*OK* orthokeratology, *SP* spectacle

### Axial length growth in ortho-k and spectacle groups

**Fig. 3:**
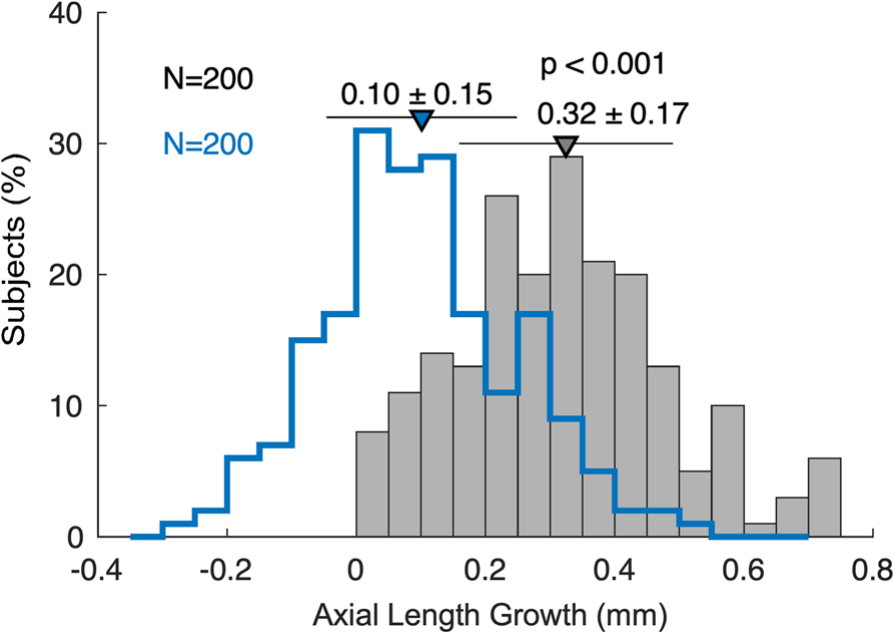
Histogram showing axial length growth of children in the ortho-k (blue line) and spectacle groups (gray line). Data are expressed as the mean ± SD

Figure 3 shows that compared to the control, children wearing orthokeratology demonstrated significantly smaller ALG over one-year treatment (mean ALG in the ortho-k group: 0.1 ± 0.15 mm; vs. mean ALG in spectacle: 0.32 ± 0.17 mm, *p* < 0.001).

### Distribution of RCRP, treatment zone decentration, and SE

**Fig. 4:**
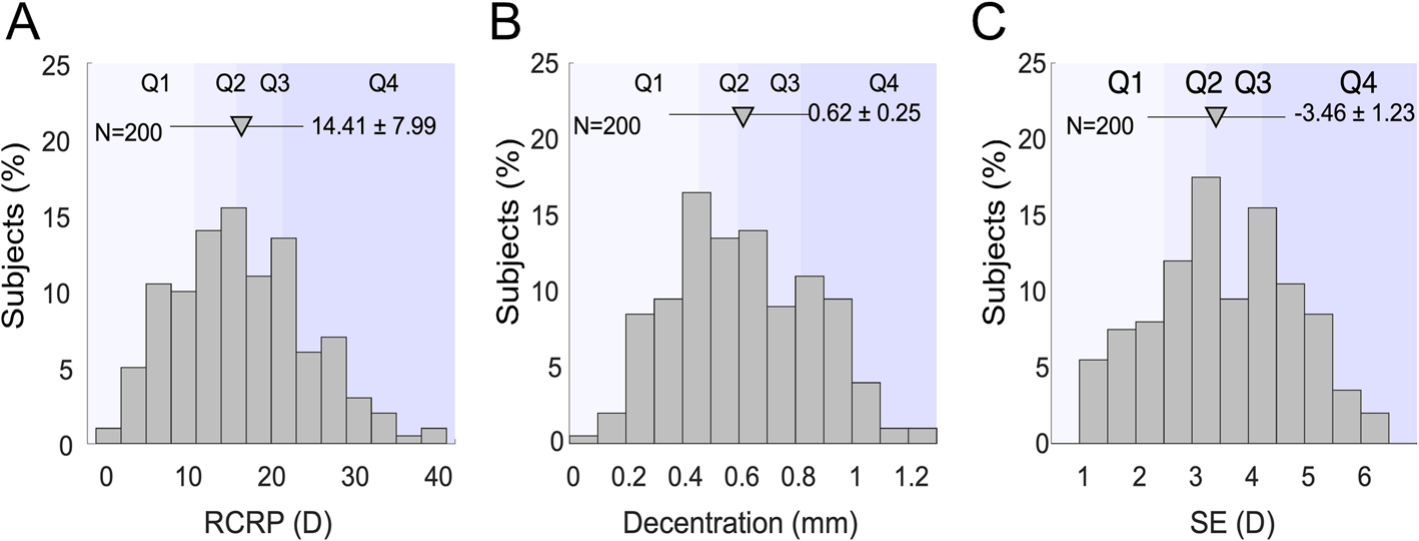
Histogram showing the distribution of RCRP, TZ decentration, and SE, the triangle representing the mean values. **A**) RCRP. **B**) TZ decentration. **C**) SE

The mean RCRP was 14.41 ± 7.99 D (Fig. 4A). Most of the subjects had TZ decentration towards the inferior temporal quadrant with a mean direction of 214.26 ± 7.39 degree, and the mean amount of TZ decentration was 0.62 ± 0.25 mm (Fig. 4B). The mean TZ decentration pointed towards the temporal and inferior sides (214.26 ± 7.39 degrees, 0 as nasal, 90 as superior, 180 as temporal, and 270 as inferior). Majority of the TZ decentration were located between 150 and 270 degrees and there was no significant difference in ALG for subjects with different decentration direction (0.11 ± 0.15 mm for 150–180 degree, 0.11 ± 0.152 mm for 180–210 degree, 0.12 ± 0.13 mm for 210–240 degree, and 0.10 ± 0.17 mm for 240–270 degree; *P* = 0.905). The mean SE was − 3.46 ± 1.23 D (Fig. 4C).

### ALG vs. summed RCRP, TZ decentration, and SE

**Fig. 5:**
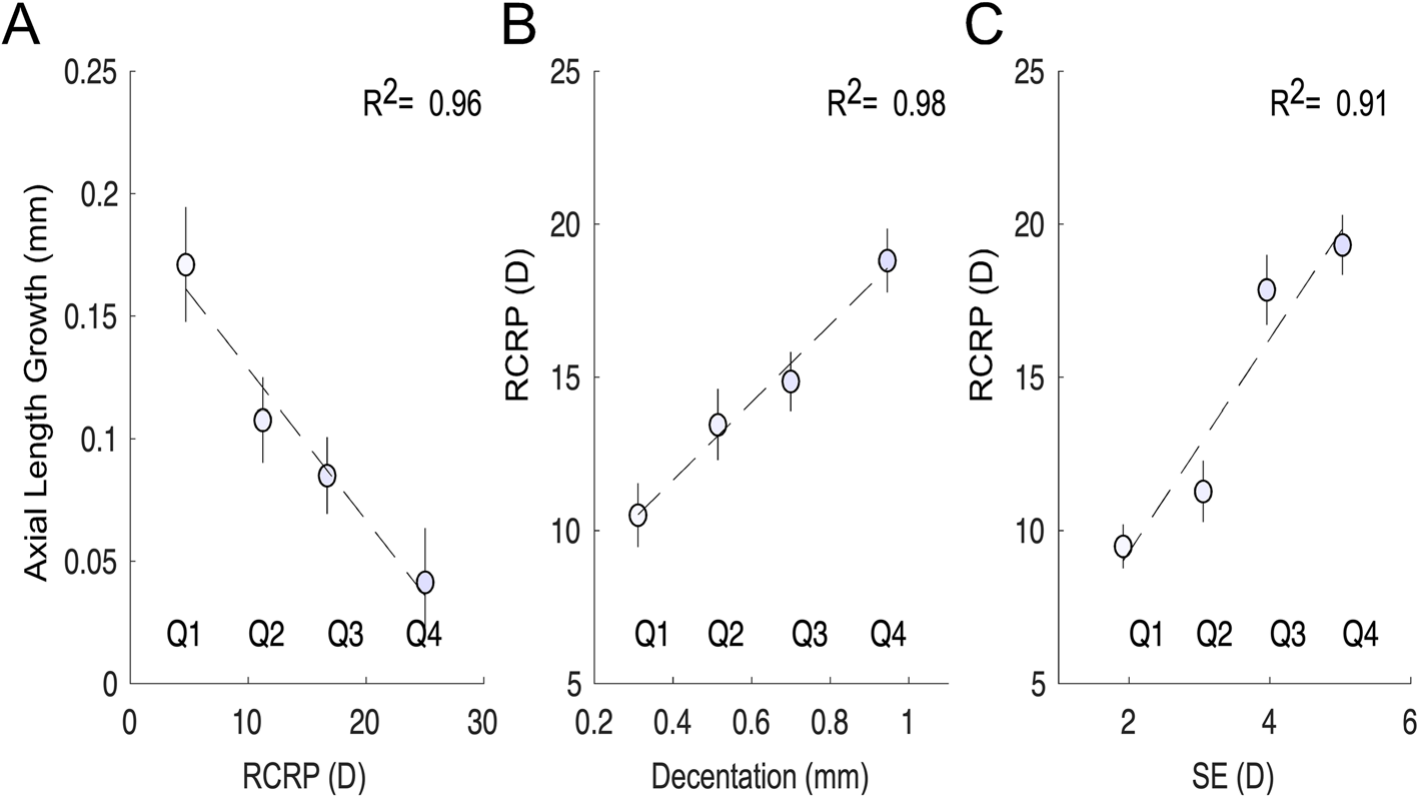
The association between ALG and RCRP, TZ decentration, and SE. **A** Correlation between ALG and RCRP. **B** Correlation between TZ decentration and RCRP. **C** Correlation between SE and RCRP

Four quartile values of RCRP were exacted (Fig. 4A), and they were significantly and negatively associated with ALG (Fig. 5A, *R*^2^ = 0.96, *p* < 0.01). TZ decentration (Fig. 5B, *R*^2^ = 0.98, *p* < 0.01) and SE (Fig. 5C, *R*^2^ = 0.91, *p* < 0.01) were both significantly and positively associated with RCRP.

### TZ Decentration and shifted RCRP profile

**Fig. 6:**
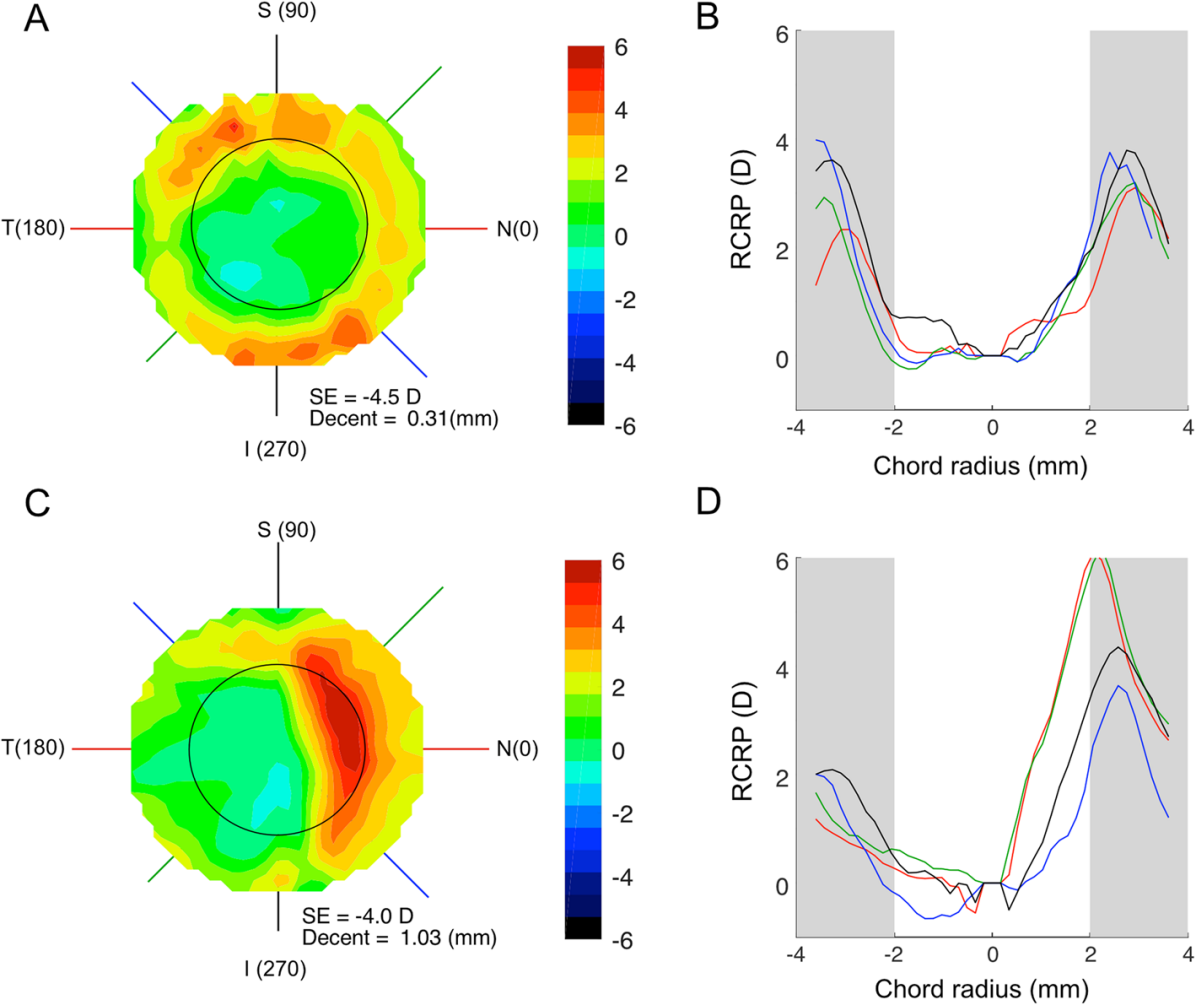
RCRP map and RCRP profiles on four major meridians. **A** RCRP map of a subject with a small TZ decentration. **B** RCRP profiles along four meridians of this subject. Red line: horizontal. Dark line: vertical. Blue and green: oblique. **C** RCRP map of a subject with a large TZ decentration. **D**. RCRP profiles along four meridians of this subject

The RCRP map of a subject with a moderate amount of TZ decentration (0.31 mm) was presented (Fig. 6A) to illustrate the effect of TZ decentration on RCRP distribution. Relative plus power evenly distributed around 360 degrees and barely invaded the central area of a radius of 2 mm (black circle). RCRP profiles along the four major meridians were basically symmetrical on each side of the cornea (Fig. 6B). In comparison, the RCRP map of a subject with a large TZ decentration (1.03 mm) was also presented (Fig. 6C). The relative plus power was mostly located on the nasal side of the cornea, and a significant portion invaded into the central area of a radius of 2 mm. The RCRP profiles were asymmetrical, with the nasal side much higher than the temporal side (Fig. 6D). The peak of the RCRP profile on the nasal (red, green line in Fig. 6D) shifted centrally, and the entire rising slope was located within the central area of a radius of 2 mm. When compared to the subject with small TZ decentration, this shifting in the subject with a large TZ decentration increases the summed RCRP within the central 2 mm area.

### TZ Decentration, the summed RCRP at different chord radii

**Fig. 7:**
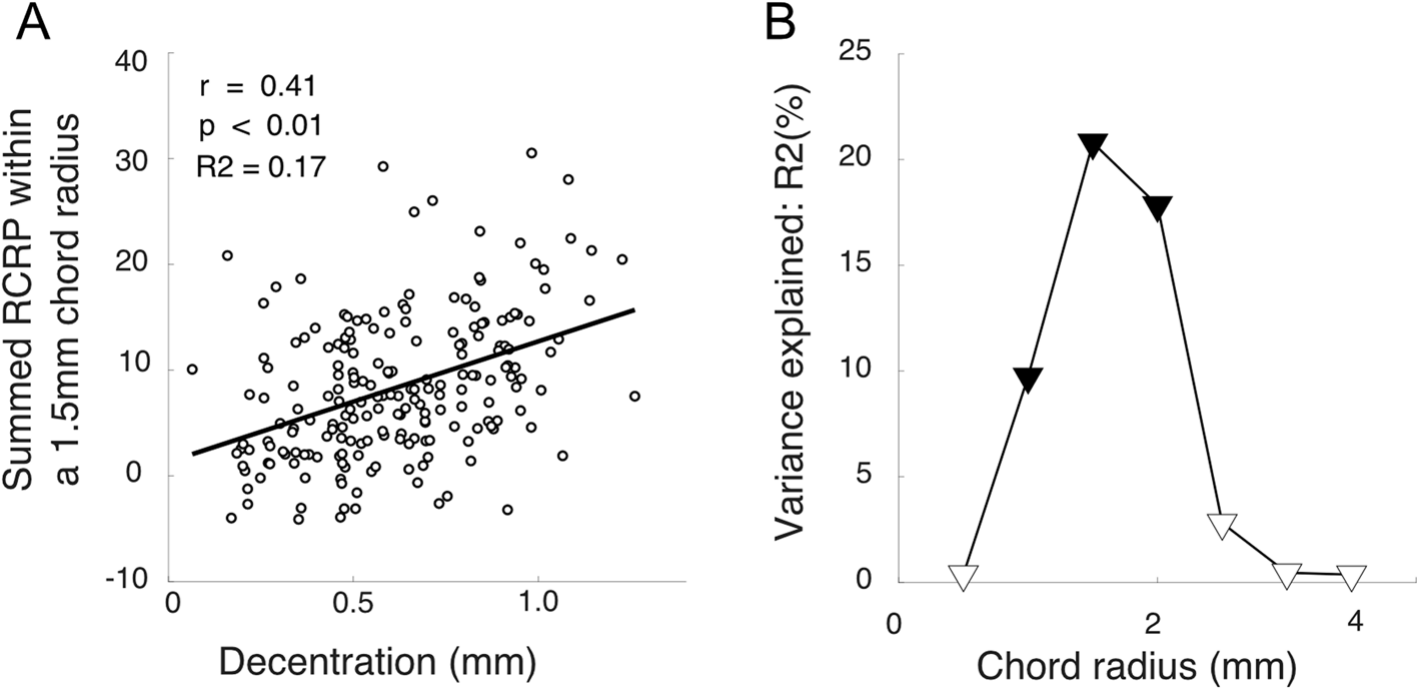
The association between TZ decentration and summed RCRP at different chord radii. **A** Correlation between TZ decentration and summed RCRP at radius 1.5 mm. **B** Simple linear regression showed that the percentage of the variance of Summed RCRP could be explained by TZ decentration at different chord radii. Solid symbols indicate statistical significance

To reveal the association between TZ decentration and summed RCRPs, we first analyzed the summed RCRPs within the central 1.5 mm radius. Simple linear correlation revealed significant correlation existed between TZ decentration and summed RCRP at this chord radius (Fig. 7A left, r = 0.41, *p* < 0.01). A similar analysis was extended to different chord radii, and the proportions of variance that could be explained were plotted against chord radii. When the chord radius was small (0.5 mm), there was no significant association. With the chord radius increasing from 1.0 mm to 2.0 mm, the association between TZ decentration and the summed RCRP became significant (Fig. 7B, solid symbols), and the proportion of variance accountable increased. When the chord radius was beyond 2 mm, the portion of variance accountable decreased, and the significance disappeared.

### SE, the summed RCRP at different chord radii

**Fig. 8:**
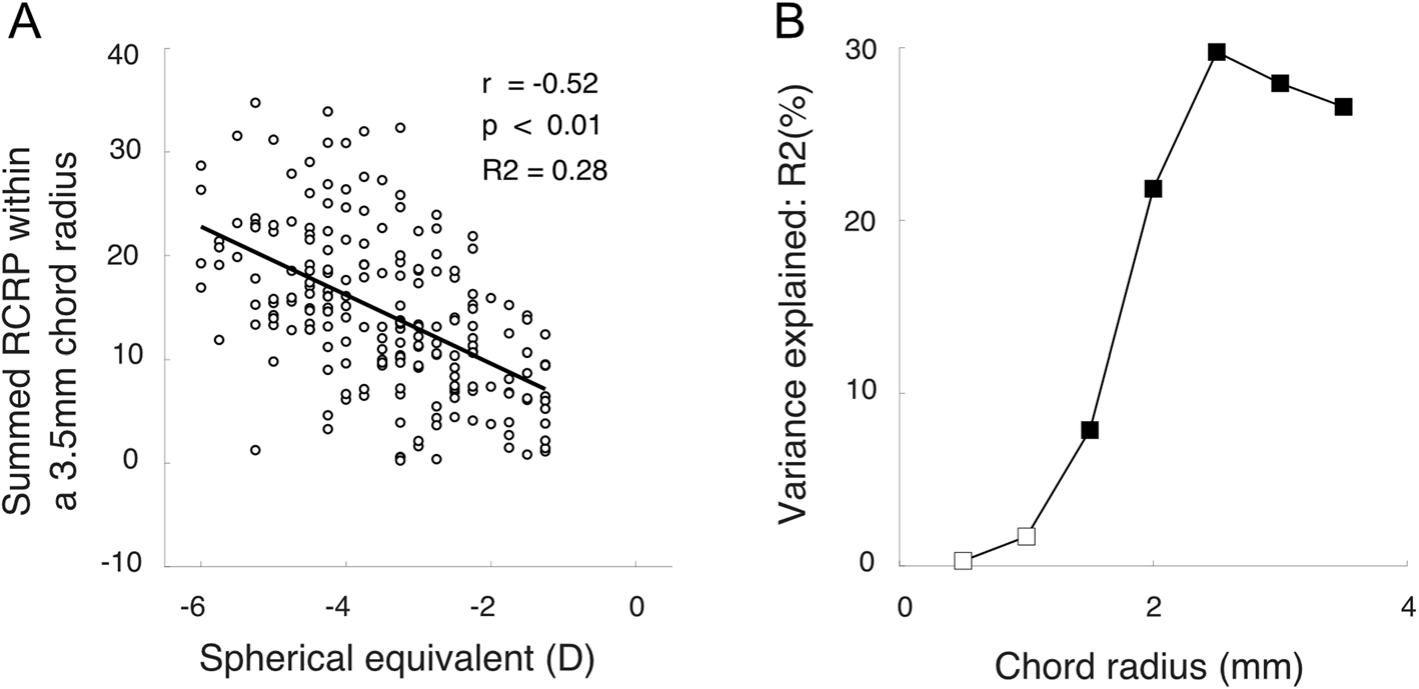
The association between SE and summed RCRP at different chord radii. **A** Correlation between SE and summed RCRP at radius 3.5 mm. **B** Simple linear regression showed that the percentage of the variance of summed RCRP could be explained by SE (black block) at different chord radii

Baseline SE was another parameter that may affect the summed RCRP. Therefore, we first analyzed the association between SE and RCRP at one chord radius of 3.5 mm. At this chord radius, SE was significantly negatively associated with summed RCRP (Fig. 8A *r* = − 0.52, *p* < 0.01). A similar analysis was extended to other chord radii. Beyond the chord radius of 1.5 mm, the association between SE and summed RCRP were all significant (solid symbols in Fig. 8B). The portion of variance accountable by SE increased and reached the peak value in 2.5 mm chord radius.

## Discussion

In this present study, we confirmed that the RCRP was negatively associated with ALG. Then we revealed a strong association between treatment zone decentration and summed RCRP in myopic children treated with orthokeratology in the univariate regression. TZ decentration was significantly positively associated with summed RCRP within 1-2 mm chord radii. SE was significantly negatively associated with summed RCRP with chord radii beyond 1 mm.

### Treatment zone Decentration amount

Many factors may contribute to the TZ decentration, such as lens fitting, lens diameter, corneal astigmatism, and eye movement during sleeping [21–23]. Traditionally, the guideline for using orthokeratology to correct visual acuity encourages clinicians to pursue a bullseye pattern in the lens fitting. The purpose of doing so is to minimize the discomfort associated with optical aberrations caused by TZ decentration. However, visual acuity correction mainly ensures a clear image in the central retinal. For retarding myopia progress, the more relevant issue is the peripheral retina defocus [24–26] and aberrations [27–29](especially spherical aberrations and vertical coma). Whether a perfectly centered OK lens is optimal for retarding myopia progress has been challenged by recent studies [6, 7]. In our previous study [8] with 352 children, the amount of TZ decentration was 0.52 ± 0.22 mm (range 0.05–1.24 mm), agreed with the current study with 200 children(0.62 ± 0.25 mm, range 0–1.2 mm). Our results are in line with the previous reports. Li et al. reported a mean TZD of 0.68 ± 0.35 mm (range 0.05–1.49 mm) from a study of 106 subjects [30], and Chen et al. reported a mean TZD of 0.72 ± 0.26 mm (range 0–1.34 mm) [23].Chen et al. reported a mean TZD of 0.64 ± 0.38 mm (range 0.13 to 1.78 mm) [6]. In the guidelines for refractive surgery, the treatment zone decentration greater than 1 mm was usually defined as severe decentration [31]. Only about 10% of the patients belong to severe decentration in our current study, which was lower than the number reported by Chen et al. (17.82%) [6] and Hiraoka’s study(33%) [11]. The discrepancy was possibly due to the difference in lens design and initial corneal power. It is essential to keep in mind that we are not suggesting an active pursuit of a lens decentration when interpreting our findings in clinical practice. Excessive TZ decentration can cause visual distortions, ghosting, visual fatigue, and other discomforts [11, 32]. It may also lead to corneal indentation and epithelium staining. The lens may adhere to the cornea and cause hypoxia in the cornea [33]. Therefore, deliberate, intentional decentration of the TZ is not recommended in clinical practice.

### Treatment zone decentration and RCRP spatial distribution

Recent studies demonstrated that the spatial distribution of the RCRP, rather than a simple summed value, is more informative for understanding the myopia retardation effect. Several new indexes have been proposed. One is X50, the radius to the center where the RCRP profile reaches the half peak. It captures the shifting of the positive RCRP towards the center well. YangXY et al. [13] demonstrated that a smaller X50 is significantly associated with smaller ALG in children wearing orthokeratology lenses with different peripheral curves. FanJ et al. [34] reported that contact lenses with smaller Half_x induce RCRPS closer to the corneal center may exert better myopia control. The other is corneal asymmetry [9, 35] which could be well quantified by the amplitude of a sinewave running one cycle over 360 degrees around the cornea. More recently, Zhang et al. found that a more aspheric treatment zone had less axial length growth in children with ortho-k treatment for a year [19]. The findings from the current study agreed well with these two indexes. TZ decentration caused the RCRP to shift towards the center and pushed the peaks higher on one side of the cornea. This increased corneal asymmetry and led to a smaller X50. We conjecture that the combination of those changes dramatically increases the chance that a region in the mid-peripheral retina to have a sufficient amount of myopic defocus for retarding myopia progress.

### Treatment zone decentration and summed RCRP

The present study revealed how TZ decentration could lead to an increased RCRP sum. As shown in Fig. 6, which assumes a 2 mm chord radius, a TZ decentration pushes the reverse zone with positive RCRP towards the center and enters the summation window on one side of the cornea. On the other side of the cornea, the rising part of the RCRP was pushed away from the center and only left a flat portion in the summation window. The net effect was a greater summed RCR*P* value in children with greater TZ decentration. This was further confirmed in Fig. 7A, which showed that the positive correlation between TZ decentration and the summed RCRP was significant when the chord radius was within 1 to 2 mm, with the peak at 1.50 mm (Fig. 7B). At chord radius beyond 2.5 mm, the association between summed RCRP and TZ decentration became insignificant. Another factor that affected the summed RCRP was SE [9]. However, the association between summed RCRP and SE was only significant when the chord radius became larger than 1 mm (Fig. 8B). This is the first study that distinguishes the effects of SE and TZ decentration on summed RCRP.

### TZ decentration and lens design

The effect of TZ decentration on ALG should not be considered alone. It should be considered in combination with the orthokeratology lens design. One key parameter is the back optical zone diameter (BOZD). A recent study [36] has suggested that a BOZD 5.5 mm is more effective than a lens with a BOZD of 6.5 mm in retarding myopia progress. For a child wearing a lens with a large BOZD, such as 6.2 mm, TZ decentration is effectively reducing the BOZD size on one side of the cornea. Therefore, our finding is consistent with the finding that prefers a smaller BOZD. We speculate that the correlation between TZ decentration and ALG in children wearing a lens with small BOZD may not be significant. Another important consideration is the toric vs. spherical design orthokeratology lens for children with substantial corneal astigmatism. In clinic practice, the corneal elevation difference between the chords of 7 and 9 mm (the first alignment curve of an orthokeratology lens mostly likely falls) is larger than 30um [37]. The spherical lens tends to be unstable in such patients, and under-corrected astigmatism, toric design orthokeratology lens would be suggested. The relationship between lens decentration and corneal elevation asymmetry at an 8-mm chord was confirmed by a study showing that both direction and amount of lens decentration were influenced by paracentral corneal asymmetry [23]. This indicates that the toric fitting technique improves lens-fitting stability in eyes with greater paracentral corneal elevation difference. Although there is a correlation between TZ decentration and myopia control in previous studies, it does not mean that the orthokeratology lens can be decentration deliberately. Therefore, a toric design lens is still very important for fitting the astigmatic cornea, even if the toric lens will reduce the occurrence of treatment zone decentration.

### Advantages and limitations of the present study

Our study has advantages when compared to previous studies. First, the influence of the distribution and morphology of RCRP caused by TZ decentration is not explicitly explained in previous studies. Second, the large sample size of the present research is several times larger than in previous studies, increasing the strength of the conclusions. Despite these improvements, there are several limitations of the current study. First, we suggested retinal myopic defocusing as a potential mechanism, but we did not directly measure retina defocusing in this study. Second, we did not measure subjects’ accommodation response after the orthokeratology treatment. Third, we did not measure subjects’ pupil sizes during the daytime, even though we speculated that TZ decentration might shift the reverse zone into the pupillary area as a potential mechanism.

## Conclusion

For myopic children undergoing orthokeratology, the TZ decentration is significantly associated with summed RCRP. It may be one of the possible reasons why TZ decentration is beneficial to myopia retardation.

## Data Availability

The datasets used and/or analyzed during the current study are available from the corresponding author on reasonable request.

## Abbreviations

TZ: Center treatment zone
RCRP: Relative corneal refractive power
SE: Spherical equivalent
BOZD: Back optic zone diameter
